# Effect of Electroacupuncture Treatment at Dazhui (GV14) and Mingmen (GV4) Modulates the PI3K/AKT/mTOR Signaling Pathway in Rats after Spinal Cord Injury

**DOI:** 10.1155/2020/5474608

**Published:** 2020-01-21

**Authors:** Ke Li, Juntong Liu, Liangyu Song, Wei Lv, Xi Tian, Zhigang Li, Suhua Shi

**Affiliations:** ^1^School of Acupuncture-Moxibustion and Tuina, Beijing University of Chinese Medicine, Beijing 100029, China; ^2^Department of TCM, Chaoyangmen Community Health Service Center, Beijing 100010, China; ^3^Department of TCM, Beijing City Chaoyang District Shuangqiao Hospital, Beijing 100121, China; ^4^Department of Medical Image, The Third Affiliated Hospital of Beijing University of Chinese Medicine, Beijing 100029, China; ^5^Department of Rehabilitation, The Third Affiliated Hospital of Beijing University of Chinese Medicine, Beijing 100029, China

## Abstract

Electroacupuncture (EA) is widely recognized as clinical treatment of spinal cord injury (SCI). The purpose of this study is to elucidate whether and how the PI3K/AKT/mTOR signaling pathway plays any role in EA treating SCI. Rats were randomly divided into four equal groups: Control Group, Sham-operation Group, Model Group, and EA Group, then further randomly divided into the following subgroups: 1-day (*n* = 12), 1-day rapamycin (*n* = 6), 14-day (*n* = 18), and 28-day (*n* = 18). A rat model of SCI was established by a modified Allen's weight-drop method. In the EA Group, rats were stimulated on Dazhui (GV14) and Mingmen (GV4) for 20 min by sterilized stainless steel needles. In the EA Group, the Basso, Beattie, and Bresnahan locomotor rating scale showed obvious improved locomotor function, and hematoxylin-eosin staining and magnetic resonance imaging showed that the histological morphology change of injured spinal cord tissue was obviously alleviated. Also, blocking spinal mTOR by injection of rapamycin showed that mTOR existed in the injured spinal cord, and EA could significantly activate mTOR in SCI rats. And immunohistochemistry and western blot analysis on the PI3K/AKT/mTOR signaling pathway showed that levels of PI3K, AKT, mTOR, and p70S6K in the injured spinal cord tissue were greatly increased in the EA Group, while the levels of PTEN and caspase 3 were decreased. The present study suggests that EA could affect cell growth, apoptosis, and autophagy through the PI3K/AKT/mTOR signaling pathway.

## 1. Introduction

Patients with spinal cord injury (SCI) usually have long-term disability causing loss of working capacity and daily living activities, which need chronic or even sometimes life-long medical care [1, 2]. SCI, an extremely serious type of physical trauma observed in clinics, has two complex temporospatial pathological phases: primary injury is always caused by trauma, whose main reasons are traffic accidents, architectural engineering, and sports or athletic events; secondary injury is instigated by the initial trauma, which is the main cause of loss of regeneration function after SCI, forming necrosis cavity and reactive proliferative glial scar [3, 4]. SCI processes a series of secondary pathophysiological changes, including apoptosis, inflammation, and nerve degeneration, which are the main hindrances affecting neural regeneration and recovery after SCI [3, 5]. So, the way of reducing secondary injury limiting proliferation of the neuroglial cell is one of the hot research subjects, promoting neuron regeneration to change the above-mentioned state after SCI.

The mammalian target of rapamycin (mTOR) is a serine/threonine protein kinase, playing an important role in regulating cell metabolism, proliferation, death, and survival in many physiological processes [6]. Furthermore, compelling evidence supports the notion that activating mTOR can effectively reduce nerve tissue damage and secondary injury after SCI, including transcription, mRNA reverse transcription, translation, ribosome synthesis, autophagy, and cytoskeleton formation [7, 8]. The PI3K/AKT/mTOR (phosphoinositide-3-kinase/protein kinase B/mTOR) signaling pathway is one of the three major signaling pathways effectively affecting mTOR [9]. After PI3K activates the intracellular signaling pathway, AKT, the downstream effector, is activated [10, 11]. As the negative regulator of the PI3K/AKT/mTOR signaling pathway, phosphatase and tensin homology deleted on chromosome ten (PTEN) could inhibit the activity of PI3K and AKT [12].

Electroacupuncture (EA), in which an electrical current is applied to acupuncture needles after they have been inserted into the body, has been widely recognized as clinical treatment of SCI [13, 14]. EA can significantly alleviate and delay the pathological damage and promote the recovery of injured spinal cord nerves after SCI, especially stimulating at “Dazhui” (GV14) and “Mingmen” (GV4) [15, 16]. It has been proved that EA has beneficial effects on neuropathic pain induced by SCI through the PI3K/AKT/mTOR signaling pathway [17]. However, until now, it remains largely elusive that EA could affect cell growth, apoptosis, and autophagy through the PI3K/AKT/mTOR signaling pathway.

Therefore, the goal of this study is to verify the therapeutic effect of EA on spinal cord injury and elucidate the effect of EA on the mTOR and PI3K/AKT/mTOR signaling pathway and further clarify the mechanism of EA in improving the pathological damage after SCI.

## 2. Materials and Methods

### 2.1. Reagent and Chemicals

The modified Allen device for a model of spinal cord injury is the NYU/MASCIS impactor device from Chinese Academy of Medical Sciences and Peking Union Medical College (Beijing, China). The sterilized stainless steel needles are 0.30 mm × 25 mm from Zhongyan Taihe Medical Instrument Co. Ltd. (Beijing, China). EA apparatus is the HANS-LH202 nerve stimulator from Beijing Huawei Industrial Development Co. Ltd. (Beijing, China). Agilent 7.0 T small animal magnetic resonance imaging equipment was from Agilent Technologies Inc. (Palo Alto, CA, US). Nikon Biological Microscope Eclipse Ci-S and Nikon Digital Sight Camera DS-U3 were from Nikon Corporation (Tokyo, Japan). Image-Pro Plus 6.0 software was from Media Cybernetics, Inc. (Bethesda, MD, US).

Sodium pentobarbital and H_2_O_2_ were from Sinopharm Chemical Reagent Co., Ltd. (Shanghai, China). Gentamicin, sodium chloride, paraformaldehyde, and ethanol were from Henan Runhong Pharmaceutical Co., Ltd. (Henan, China). Rapamycin was from Selleck Chemicals (Houston, TX, USA). Isoflurane inhalation was from RWD Life Science Co., Ltd. (Shenzhen, China). Hematoxylin and eosin were from Beijing Zhongke Wanbang Biotechnology Co., Ltd. (Beijing, China). BSA, TBST, ECL Western Blotting Substrate Kit, and SDS-PAGE Gel Kit were from Beijing Solarbio Science & Technology Co., Ltd. (Beijing, China). Antibodies to caspase 3, mTOR, p-mTOR, p70S6, p-p70S6, PI3K, p-PI3K, PTEN, and AKT were purchased from Cell Signaling Technology, Inc. (Beverly, MA, US). PBS solution and diaminobenzidine (DAB) solution were from Wuhan Boster Biological Technology Co., Ltd. (Wuhan, China). The antibody for goat-anti-rabbit IgG and reduced glyceraldehyde-phosphate dehydrogenase (GAPDH) were purchased from Santa Cruz Biotechnology, Inc. (Santa Cruz, CA, US). RIPA Lysis Buffer and BCA Protein Assay Kit were from Thermo Fisher Scientific, Inc. (Fair Lawn, NJ, US). The PVDF membrane was obtained from Millipore (Billerica, MA, US). Skim milk was from Inner Mongolia Yili Industrial Group Limited by Share Ltd. (Inner Mongolia, China).

### 2.2. Animals and Groups

The protocol for the animal study was approved by the Institutional Animal Care and Use Committee of Beijing University of Chinese Medicine, China, and all the efforts were made to ameliorate suffering of animals. All 13-week-aged male Sprague-Dawley rats were purchased from SPF (Beijing) Biotechnology Co., Ltd. (Beijing, China). The certificate number was SCXK (Beijing) 2016-0002. All of the rats (body weight range 200–240 g) were maintained under the standard laboratory conditions (25 ± 3°C, 45 ± 10% relative humidity, and 12-hour light/dark cycle), with access to standard chow and water ad libitum. All rats were randomly divided into four groups: Control (C) Group (*n* = 54) without any intervention, Sham-operation (S) Group (*n* = 54) which received only laminectomy, Model (M) Group (*n* = 54) which received SCI induction at the T10 spinal segment, and Electroacupuncture (EA) Group (*n* = 54) which received SCI induction at the T10 spinal segment and EA intervention at GV14 and GV4. Rats in the above four groups were randomly divided into the following subgroups: 1-day (*n* = 12), 1-day rapamycin (*n* = 6), 14-day (*n* = 18), and 28-day (*n* = 18).

After being handled as the Control Group, Sham-operation Group, Model Group, and EA Group required, the rats of 1-day, 14-day, and 28-day subgroups were euthanized, respectively, on the 1st, 14th, and 28th days, while rats of the 1-day rapamycin subgroup were injected with rapamycin for 3 consecutive days before being handled as required by the four groups and then were euthanized on the 1st day.

### 2.3. SCI Model

**Figure 1:**
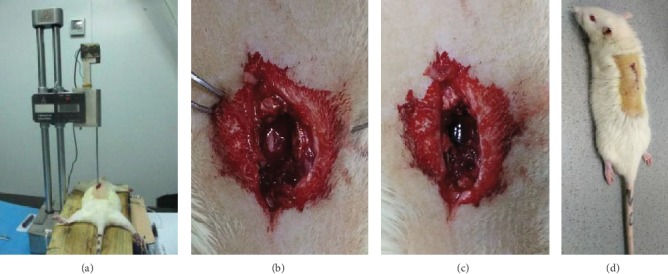
(a) The modified Allen device for a model of spinal cord injury; (b) before SCI on T10; (c) after SCI on T10; (d) SCI rat.

A rat model of SCI was established using the established methods. The rats of the Model Group and EA Group were injected with 3% sodium pentobarbital (2.0 ml/kg) anesthesia and bound in prone position on a cork platform. After anesthesia, the skin was incised along the midline of the dorsum to expose 2.5 cm median longitudinal incision with the thoracic (T) 10 spinous process as the center on the vertebral column (Figure 1(b)). After laminectomy of the whole T10 lamina to expose an approximately 10 mm segment of the spinal cord, put the rats on the platform of the modified Allen device (10 g/cm, impactor pestle diameter: 2 mm) (Figure 1(a)). The impactor pestle was subsequently dropped from a height of 70 mm to the exposed spinal cord with body convulsive tremor and tail convulsive acts to have spinal cord ischemia and edema around the wound (Figure 1(c)). After rinsing the wound with sterile saline and placing absorbable gelatin sponge, the muscles and skin were sutured in layers. All SCI rats, awakened after surgery, had lower limb paralysis (Figure 1(d)). They were treated with an intraperitoneal injection of gentamicin at a dose of 2000 U/day for a week and underwent manual urinary bladder emptying three times a day.

The rats of the Sham-operation Group only underwent laminectomy which had the same steps as in the above. Awakened after surgery, the rats were not paralyzed. They also were treated with an intraperitoneal injection of gentamicin at a dose of 2000 U/day for a week and had no urinary retention.

### 2.4. EA Stimulation

EA was performed each day on the EA group. The rats were bound in prone position, and Dazhui (GV14, located on the posterior midline below the spinous process of the seventh cervical vertebra) and Mingmen (GV4, located on the posterior midline below the spinous process of the second lumbar vertebra) were stimulated by 0.30 mm × 25 mm sterilized stainless steel needles at an angle of 15-45 degrees, with a depth of 0.5-0.7 cm. The needles were connected to the electrodes of the HANS-LH202 nerve stimulator with a frequency of 2 Hz and an intensity of 1 mA for 20 minutes. The rats of the EA Group were treated with EA for the first time 2 hours after awakening from anesthesia and then once per day until they were euthanized at the appointed time. The rats of the 1-day group were given the last treatment before euthanasia. In the EA stimulation procedure, all rats maintained relatively comfortable states.

The rats of the Control Group, Sham-operation Group, and Model Group were only bound in prone position for 20 minutes when the EA group received treatment.

### 2.5. Intervention of Rapamycin

The rats of 1-day rapamycin (*n* = 6) from the Control Group, Sham-operation Group, Model Group, and EA Group received rapamycin (3.0 mg/kg) by intraperitoneal injection for 3 consecutive days before the experiment. The rest of the procedures were implemented in accordance with the requirements of the Control Group, Sham-operation Group, Model Group, and EA Group.

### 2.6. Behavioral Testing

Basso, Beattie, and Bresnahan (BBB), which is a 21-point system hind limb locomotor rating scale, was used to evaluate the locomotor function of hind limbs in rats. Before euthanasia, the rats of 1-day (*n* = 12), 14-day (*n* = 18), and 28-day (*n* = 18) subgroups from the Control Group, Sham-operation Group, Model Group, and EA Group were assessed by two observers who were blinded to the grouping, using the locomotor rating scale ranging from 0 point indicating complete hind limb paralysis to 21 points denoting completely normal locomotor function. The scores of all the rats were assessed after modeling and before euthanasia, and eventually, the average was taken.

### 2.7. Magnetic Resonance Imaging (MRI)

The rats of 1-day (*n* = 6), 14-day (*n* = 6), and 28-day (*n* = 6) subgroups from the Control Group, Sham-operation Group, Model Group, and EA Group were examined by magnetic resonance imaging after BBB testing. MRI was performed on Agilent 7.0 T small animal magnetic resonance imaging equipment by a professional operator. The rats were anesthetized with 2% isoflurane inhalation and monitored continuously during the imaging session. Anesthetized rats were placed prone in a special coil of the equipment, then examined by MRI centered on the cervical spine of the rat. The imaging parameters were as follows: T2-weighted imaging (T2WI) has repetition time (TR) = 3800 ms, echo time (TE) = 72 ms, acquisition time (TA) = 12 min 21 s, slice thickness = 1 mm, field‐of‐view (Fov) = 60 mm × 60 mm, and acquisition matrix = 256 × 256. Diffusion tensor imaging (DTI) has TR = 2000 ms, TE = 120 m, TA = 3 min 38 s, slice thickness = 3 mm, slice gap = 0.12 mm, Fov = 60 mm∗60 mm, matrix = 128 × 128, and *b* value = 1000 s/mm^2^.

All images, reconstructed in the sagittal plane, were imported into Image-Pro Plus 6.0 software to observe the histology structure. Compared with the spinal cord tissue of the Control Group, intensity thresholds for both hypointense and hyperintense were determined. The thresholds were applied to the spinal cord segment with the T10 as the center extended to T8-T12. The inflammatory region, which was in the hyperintense region on T2WI in each image, was observed and calculated.

### 2.8. Hematoxylin-Eosin (HE) Staining

The rats of 1-day (*n* = 6), 14-day (*n* = 6), and 28-day (*n* = 6) subgroups from the Control Group, Sham-operation Group, Model Group, and EA Group were injected with 3% sodium pentobarbital (2.0 ml/kg) anesthesia and bound in supine position on a cork platform. After thoracotomy, rats were transcardially perfused with 0.9% sodium chloride and then 4% paraformaldehyde until liver whitening and tail stiffening. The spinal cord segment, which was surgically obtained 1 cm centered on the T10 segment, was kept in 4% paraformaldehyde for 24 hours. The spinal cord segment, dehydrated with ethanol and embedded with paraffin, was serially sliced into 5 *μ*m coronal sections which were stained with hematoxylin and eosin targeting the T10 segment. 5 sections in each segment were randomly selected to assess the histopathologic changes, and the sections were observed at 40x, 100x, and 200x with Nikon Biological Microscope Eclipse Ci-S and Nikon Digital Sight Camera DS-U3. And to quantify the cystic cavity, all 40x HE images were imported in Image-Pro Plus 6.0 software to calculate the ratio of the cystic cavity tissue area to the cystic cavity area.

### 2.9. Immunohistochemical Staining

The 1 cm spinal cord segments centered on the T10 segment, of 1-day (*n* = 6), 14-day (*n* = 6), and 28-day (*n* = 6) subgroups from the Control Group, Sham-operation Group, Model Group, and EA Group, were serially sliced into 5 *μ*m coronal sections after being dehydrated and embedded. 5 sections in each segment, which were randomly selected, were kept in 5% BSA at 37°C for 20 minutes for blocking, after in 3% H_2_O_2_ at 37°C with 10-minute incubation to quench endogenous peroxidase activity, and then in PBS solution for 10 minutes. The following antibodies were used as the primary antibody: caspase 3 (1 : 200), p-mTOR (1 : 200), and p-PI3K (1 : 200), to incubate at 4°C for the night. The next day, after being rinsed three times in PBS (pH = 7.4), the sections were incubated with goat-anti-rabbit IgG at 37°C for 20 minutes, then rinsed in PBS again, with SABC at 37°C for 20 minutes. The sections were rinsed with PBS and placed into diaminobenzidine (DAB) solution for 5 minutes after being rinsed for another time with PBS. After being redyed, the spinal cord sections were dehydrated and were observed at 200x with Nikon Biological Microscope Eclipse Ci-S and Nikon Digital Sight Camera DS-U3.

All the sections were imported in Image-Pro Plus 6.0 software to analyze the immunofluorescence-positive products in each group. The integrated optical density (IOD) of the positive reaction site was recorded and calculated, which represents the strength of staining signals as measured per positive pixel.

### 2.10. Western Blot (WB)

After being ground into tissue homogenate, each 1 cm spinal cord segment, from the rats of all subgroups (*n* = 6) from the Control Group, Sham-operation Group, Model Group, and EA Group, was added to 50 *μ*l RIPA Lysis Buffer. Samples were centrifuged at 10000 r/min for 20 minutes at 4°C. The protein content was determined by the BCA protein quantitative method. For electrophoresis, protein samples (40 *μ*g each) were dissolved in the sample buffer and heated to 100°C for 5 minutes. After quantification, the protein samples were separated by 10% SDS-PAGE and transferred to PVDF membranes. The membranes were blocked in Tris-buffered saline-Tween (TBST) containing 5% skim milk for 1 hour, followed by incubation at 4°C overnight with the specific antibodies: caspase 3 (1 : 1000), mTOR (1 : 1000), p-mTOR (1 : 1000), p70S6 (1 : 2000), p-p70S6 (1 : 2000), PI3K (1 : 2000), p-PI3K (1 : 1000), PTEN (1 : 2000), and AKT (1 : 3000). After being stained, the membranes were incubated with HRP goat-anti-rabbit IgG (1 : 5000). Proteins were visualized by ECL for 5 minutes to get the bands. Bands were quantified using Image-Pro Plus 6.0 image analysis software. Reduced glyceraldehyde-phosphate dehydrogenase (GAPDH) served as an internal control. The gray values of target proteins were divided by those of GAPDH to correct the error, which resulted due to the relative content of the target protein in the sample.

### 2.11. Statistical Analysis

All the data was analyzed using Microsoft Excel 2010 and SPSS Statistics 17.0 (SPSS, USA). The statistical analysis was performed using the SPSS software, version 17.0 (SPSS, Inc., Chicago, IL, USA), and the data were expressed as the mean ± standard deviation. A one-way ANOVA was used after the normal distribution and homogeneity of variance were confirmed. For the nonnormally distributed data or for data with heterogeneous variance, a nonparametric test was used. The LSD method was applied for pairwise comparisons of the western blot results. Statistical significance was set to *p* < 0.05, and high statistical significance was set to *p* < 0.01.

## 3. Results

### 3.1. Effects of EA on Locomotor Function after Spinal Cord Injury

**Figure 2:**
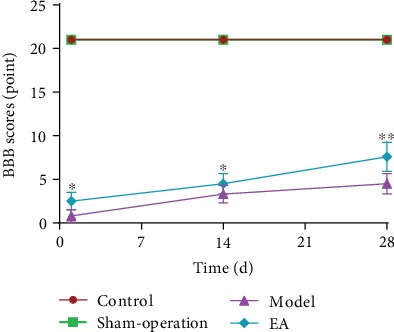
The result of behavioral testing in the Control Group, Sham-operation Group, Model Group, and EA Group on the 1st (*n* = 12), 14th (*n* = 18), and 28th (*n* = 18) days. ^∗^*p* < 0.05 and ^∗∗^*p* < 0.01, as compared with the Model Group.

Compared with the Control Group and Sham-operation Group, BBB scale scores in the Model Group and EA Group were significantly lesser at all time points (*p* < 0.01). Compared with the Model Group, BBB scale scores in the EA Group were high in 1-day and 14-day subgroups (*p* < 0.05) and significantly greater in the EA Group in the 28-day subgroup (*p* < 0.01) (Figure 2).

### 3.2. Effects of EA on Morphology Changes after Spinal Cord Injury

**Figure 3:**
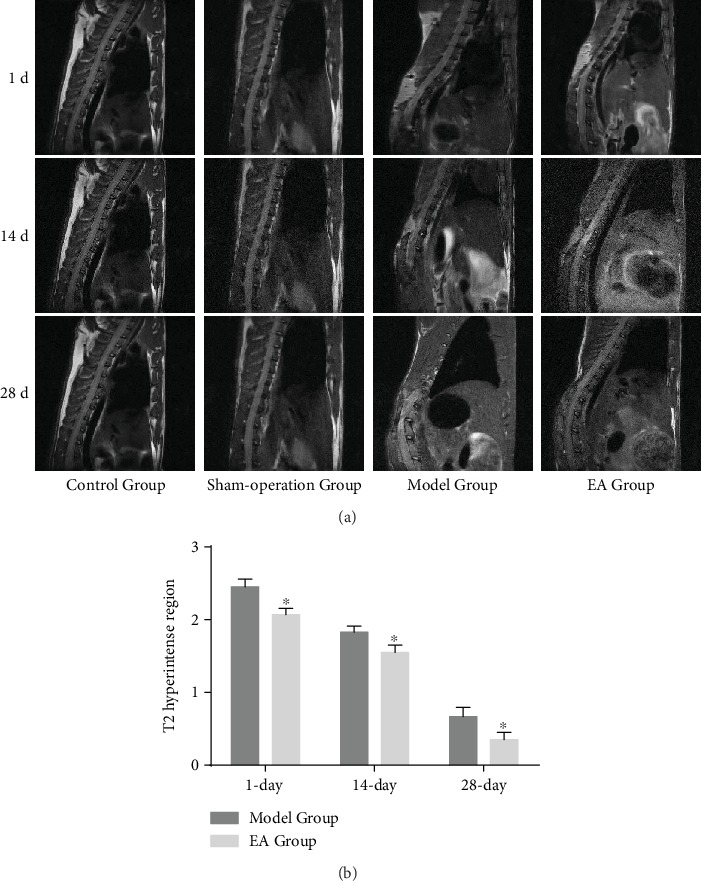
(a) Serial MRIs of the Control Group, Sham-operation Group, Model Group, and EA Group on the 1st, 14th, and 28th days; (b) the T2 hyperintense region in the Model Group and EA Group on the 1st (*n* = 6), 14th (*n* = 6), and 28th (*n* = 6) days. ^∗^*p* < 0.05, as compared with the Model Group.

MRI showed the morphology of the thoracic segment of the spinal cord in all rats, especially the T10 segment. The spinal cords of rats from the Control Group and Sham-operation Group have no obvious compression, by which MRI shows no abnormal signal. The spinal cords of rats from the Model Group and EA Group have compressive deformation forming necrosis cavity and reactive proliferative tissue, by which MRI shows that on T2WI, inflammation was in the hyperintense region which progressively decreases as time goes on (Figure 3(a)). Compared with the Model Group, MRI in the EA Group shows the more complete organizational structure, smaller volume of the T2 hyperintense region, and lower signal, which means that rats in the EA Group have less damaged tissue and inflammation (*p* < 0.05) (Figure 3(b)).

### 3.3. Effect of EA on Histological Changes in Injured Spinal Cord

**Figure 4:**
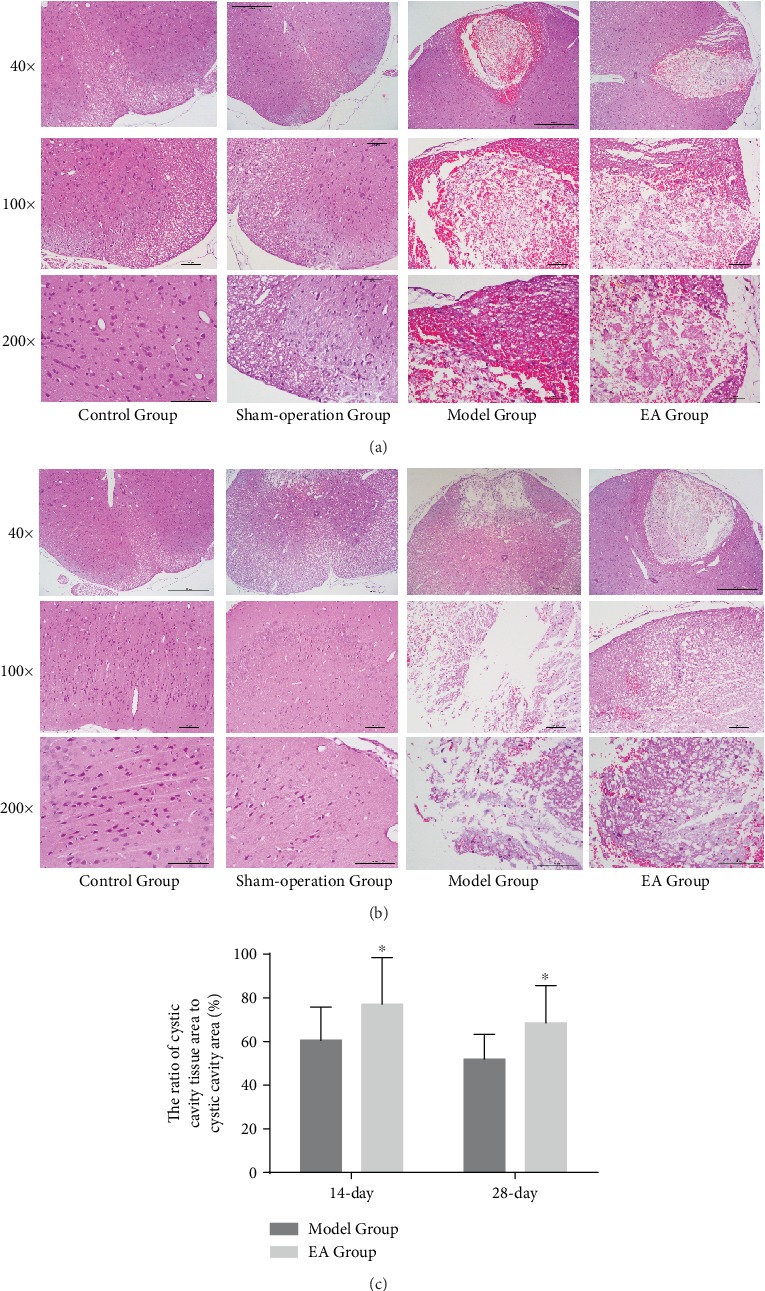
(a) Histological changes of all groups on the 14th day. (b) Histological changes of all groups on the 28th day. (c) The ratio of the cystic cavity tissue area to the cystic cavity area in the Model Group and the EA Group on the 1st (*n* = 6), 14th (*n* = 6), and 28th (*n* = 6) days. ^∗^*p* < 0.05, as compared with the Model Group.

The spinal cord tissues are normal in the Control Group and Sham-operation Group, which had a complete structure, normal gray and white matter, and regular cells. In contrast, the injured regions in the spinal cord in the Model Group and EA Group displayed disruption of structures. On day 14, the necrosis cavity was visible in the damaged region, where neuron cells were decreased largely, with blood cells and inflammatory cell infiltration. In addition, the boundary of the gray and white matter was unclear, and the neurons were swollen; pyknosis of part of the neuronal nuclei was observed (Figure 4(a)). On day 28, the necrosis cavity remained visible with less bleeding, and neuronal edema was alleviated (Figure 4(b)). Compared with the Model Group at the same time, the injured region in the spinal cord in the EA Group displayed improved tissue repair and recovery with less pathologic injury in the necrosis cavity (*p* < 0.05) (Figure 4(c)), which had less bleeding, lighter neuron cell edema, more normal neuron cells, and more dense tissue.

### 3.4. Effect of EA on mTOR in the Injured Spinal Cord

**Figure 5:**
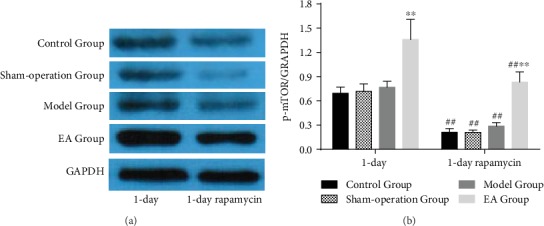
The result of p-mTOR protein expression in the Control Group, Sham-operation Group, Model Group, and EA Group from the 1-day subgroup (*n* = 6) and 1-day rapamycin subgroup (*n* = 6). ^##^*p* < 0.01, as compared with the same name group in the 1-day subgroup. ^∗∗^*p* < 0.01, as compared with the Model Group.

Compared with the Control Group, Sham-operation Group, and Model Group, the level of p-mTOR protein expression in the EA Group was significantly increased in the 1-day subgroup (*p* < 0.01) (Figure 5). The levels of p-mTOR protein expression in all groups from the 1-day rapamycin subgroup were significantly decreased compared with those in the same name group from the 1-day subgroup (*p* < 0.01) (Figure 5), which showed that the amplified activities of p-mTOR, even evoked by SCI, were significantly attenuated. And the level of p-mTOR protein expression in the EA Group from the 1-day rapamycin subgroup was still significantly increased compared with that in other groups from the same subgroup (Figure 5), which showed that EA could activate mTOR in the injured spinal cord.

### 3.5. Effect of EA on the PI3K/AKT/mTOR Signaling Pathway in the Injured Spinal Cord

#### 3.5.1. Immunohistochemical Results

**Figure 6:**
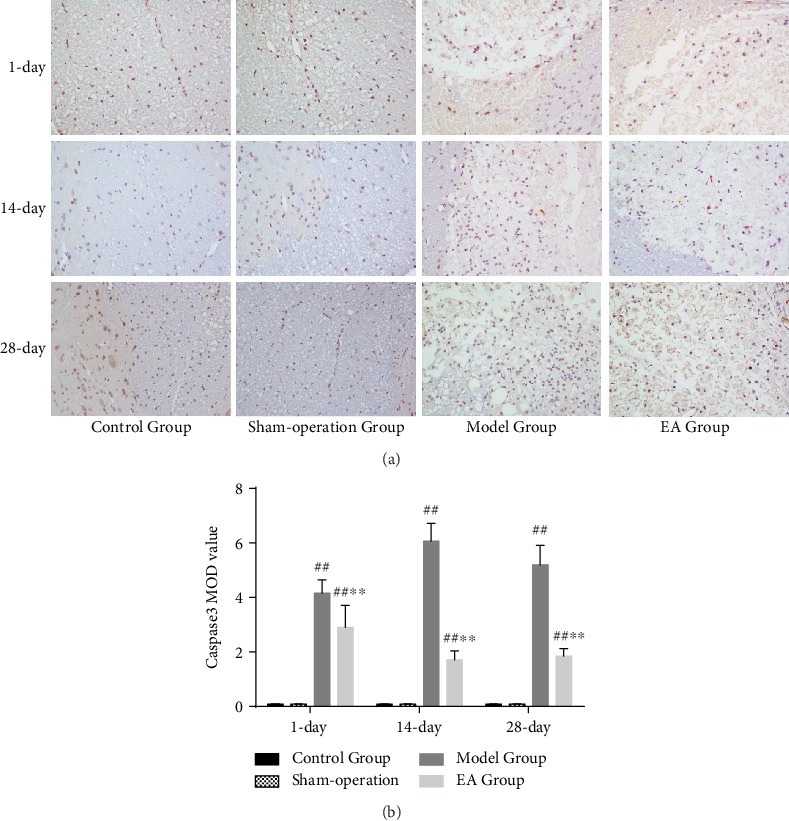
(a) The caspase 3-immunoreactive cells in the Control Group, Sham-operation Group, Model Group, and EA Group on the 1st, 14th, and 28th days (×400). (b) IOD of caspase 3 in the Control Group, Sham-operation Group, Model Group, and EA Group on the 1st (*n* = 6), 14th (*n* = 6), and 28th (*n* = 6) days. ^##^*p* < 0.01, as compared with the Control Group and Sham-operation Group. ^∗∗^*p* < 0.01, as compared with the Model Group.

**Figure 7:**
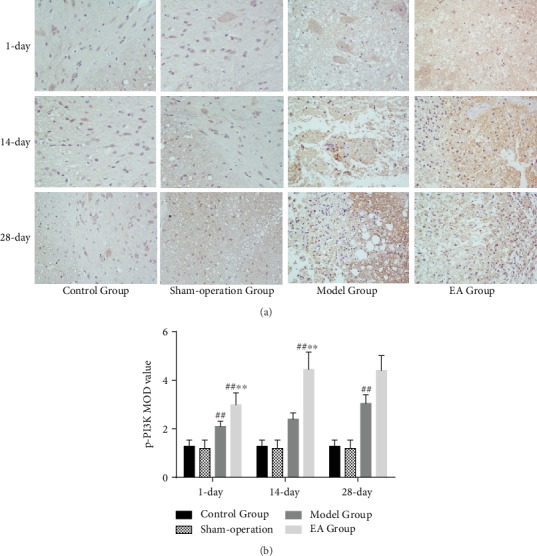
(a) The p-PI3K-immunoreactive cells in the Control Group, Sham-operation Group, Model Group, and EA Group on the 1st, 14th, and 28th days (×400). (b) IOD of p-PI3K in the Control Group, Sham-operation Group, Model Group, and EA Group on the 1st (*n* = 6), 14th (*n* = 6), and 28th (*n* = 6) days. ^##^*p* < 0.01, as compared with the Control Group and Sham-operation Group. ^∗∗^*p* < 0.01, as compared with the Model Group.

Compared with the Control Group and Sham-operation Group, the numbers of caspase 3-immunoreactive cells and p-PI3K-immunoreactive cells were increased in the Model Group and EA Group on the 1st (*p* < 0.01), 14th (*p* < 0.01), and 28th (*p* < 0.01) days. Compared with the Model Group, the numbers of caspase 3-immunoreactive cells and p-PI3K-immunoreactive cells were decreased in the EA Group on the 1st (*p* < 0.01), 14th (*p* < 0.01), and 28th (*p* < 0.01) days (Figures 6 and 7).

**Figure 8:**
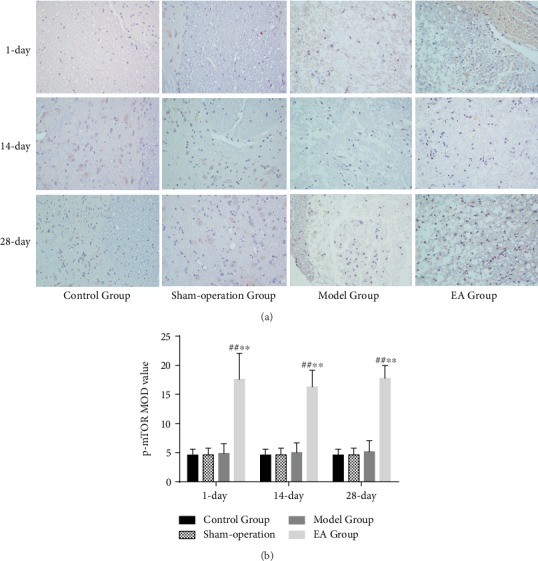
(a) The p-mTOR-immunoreactive cells in the Control Group, Sham-operation Group, Model Group, and EA Group on the 1st, 14th, and 28th days (×400). (b) IOD of p-mTOR in the Control Group, Sham-operation Group, Model Group, and EA Group on the 1st (*n* = 6), 14th (*n* = 6), and 28th (*n* = 6) days. ^##^*p* < 0.01, as compared with the Control Group and Sham-operation Group. ^∗∗^*p* < 0.01, as compared with the Model Group.

Compared with the Control Group, Sham-operation Group, and Model Group, the number of p-mTOR-immunoreactive cells was increased in the EA Group on the 1st (*p* < 0.01), 14th (*p* < 0.01), and 28th (*p* < 0.01) days (Figure 8).

#### 3.5.2. Western Blot Analysis Results

**Figure 9:**
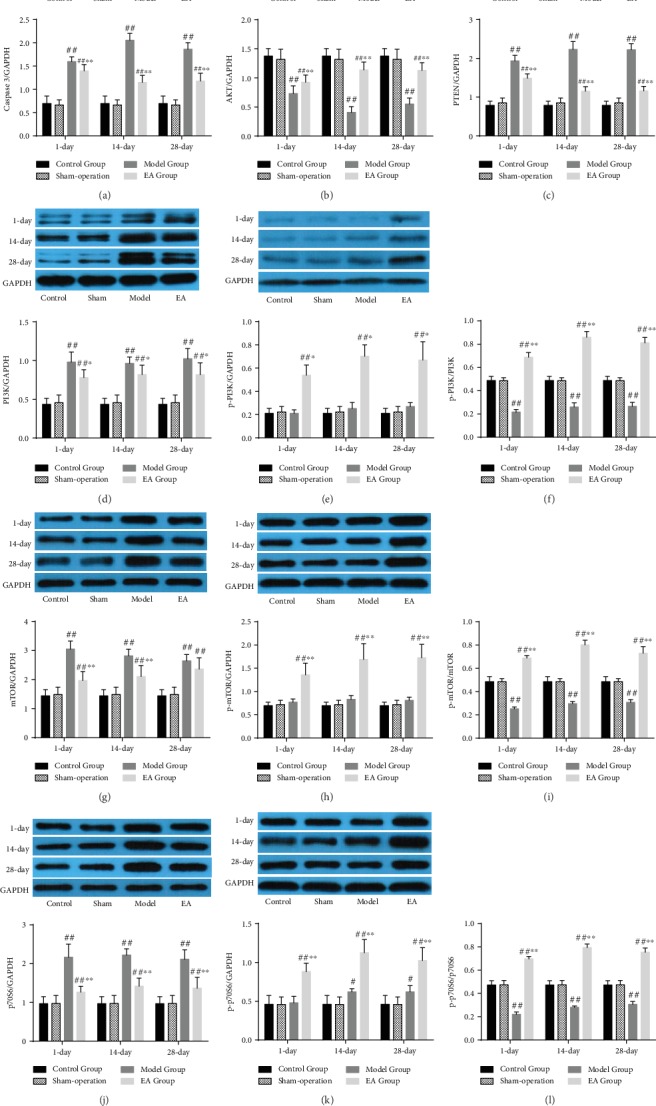
The expressions of PI3K/AKT/mTOR signaling pathway-related factors caspase 3 (a), AKT (b), PTEN (c), PI3K (d), p-PI3K (e), p-PI3K/PI3K (f), mTOR (g), p-mTOR (h), p-mTOR/mTOR (i), p70S6 (j), p-p70S6 (k), and p-p70S6/p70S6 (l) in the Control Group, Sham-operation Group, Model Group, and EA Group on the 1st (*n* = 6), 14th (*n* = 6), and 28th (*n* = 6) days. ^#^*p* < 0.05, ^##^*p* < 0.01, as compared with the Control Group and Sham-operation Group. ^∗^*p* < 0.05, ^∗∗^*p* < 0.01, as compared with the Model Group.

Compared with the Control Group and Sham-operation Group, the expressions of caspase 3, AKT, and PTEN were significantly different in the Model Group and EA Group. Compared with the Model Group, the expressions of caspase 3 and PTEN were significantly decreased in the EA Group (*p* < 0.01) (Figures 9(a) and 9(c)), and AKT was significantly increased (*p* < 0.01) (Figure 9(b)).

Compared with the Control Group and Sham-operation Group, the expressions of PI3K, mTOR, and p70S6 were significantly increased in the Model Group and EA Group. Compared with the Model Group, the expressions of PI3K, mTOR, and p70S6 were significantly decreased in the EA Group (*p* < 0.05 or *p* < 0.01) (Figures 9(d), 9(g), and 9(j)). The expressions of p-PI3K, p-mTOR, and p-p70S6 among the Control Group, Sham-operation Group, and Model Group were not significantly altered, whereas the expression levels were significantly increased in the EA Group (*p* < 0.01) (Figures 9(e), 9(h), and 9(k)). And compared with the Control Group, Sham-operation Group, and Model Group, the p-PI3K/PI3K, p-mTOR/mTOR, and p-p70S6/p70S6 ratios were significantly increased in the EA Group (*p* < 0.01) (Figures 9(f), 9(i), and 9(l)).

These results primarily showed that SCI inhibited the PI3K/AKT/mTOR signaling pathway and EA activated the SCI-inhibited PI3K/AKT/mTOR signaling pathway.

## 4. Discussions

The PI3K/AKT/mTOR signaling pathway contributes to a variety of processes, nutrition absorption, anabolism, and so on, which are crucial to mediating many aspects of cell function, including cell survival, proliferation, growth, metabolism, angiogenesis, and metastasis [10–12, 18].

PI3K is the upstream promoter of the PI3K/AKT/mTOR signaling pathway [19]. Under the effect of stress from the outside, activated by coupling with receptor tyrosine kinases (RTKs), PI3K phosphorylate PtdIns(4,5)P2 (PIP2) to form PtdIns(3,4,5)P3 (PI3P), a second messenger, which could activate AKT [10]. The phosphorylated PTEN, a negative regulator, could inhibit the PI3K/AKT signaling pathway by dephosphorylating PIP3 [12]. Activated AKT could regulate cell function by phosphorylating downstream factors such as enzymes, kinases, and transcription factors [11].

In addition, mTOR, an important PI3K/AKT downstream protein kinase, is present in mTOR complex 1 (mTORC1) and mTOR complex 2 (mTORC2) [20]. mTORC2 is related to the construction of the cell skeleton and cell movement, while mTORC1 is the main regulator of cell proliferation, apoptosis, and autophagy [21]. mTORC1 could be activated by Ras homology enriched in brain (Rheb) enrichment, and AKT could phosphorylate tuberous sclerosis complex (TSC1/2), which reduces negative regulation to Rheb enrichment from TSC1/2 [11, 22]. AKT also could activate mTORC1 by phosphorylating PRAS40 to reduce competitive binding of PRAS40 and mTORC1 [23]. mTORC1 activates downstream protein translation by phosphorylating eukaryotic transcription initiation factor 4E-binding protein 1 (4EBP1) and p70 ribosomal protein S6 kinase (p70S6K) to regulate cell proliferation, differentiation, autophagy, and apoptosis [21, 24]. The caspase family plays important roles in the process of cell apoptosis, and caspase 3 is the most critical executor [25].

The previous results show that the activity of the PI3K/AKT/mTOR signaling pathway decreases after SCI in rats, and with the increased activity of the PI3K/AKT/mTOR signaling pathway, the motor function of hind limbs and the spinal neurological function recovery are improved in rats [26–28]. And it is shown that after knocking out the PTEN gene, the activity of mTOR is increased in SCI rats, which effectively protects neurons and promotes axon regeneration [29]. It is also verified that a PI3K inhibitor, LY294002, or an mTOR inhibitor, rapamycin, could block the PI3K/AKT/mTOR signaling pathway and relate to neuropathic pain evoked by SCI [30, 31].

All the above show that the PI3K/AKT/mTOR signaling pathway has important significance for functional recovery after SCI. However, the endogenous activation mechanism of the PI3K/AKT/mTOR signaling pathway has limited effect on capability of self-reparation of neurons after SCI. Therefore, how to find the exogenous way to increase the activity of the PI3K/AKT/mTOR signaling pathway is one of important contents in the feasibility study. Effect of EA treating SCI is already confirmed in the clinical practice and recognition [13, 14]; however, whether and how the PI3K/AKT/mTOR signaling pathway plays any role in EA treating SCI are unknown.

In this study, it is testified that EA could improve anatomical and functional repair by HE staining and MRI in the injured spinal cord and locomotor function in SCI rats by BBB behavioral testing. And the result shows that, with EA treatment, the level of mTOR in the injured spinal cord is significantly increased in SCI rats, whereas injection of rapamycin could block mTOR induced by EA, indicating that mTOR and related signaling pathways exist in the injured spinal cord and EA could significantly activate mTOR in SCI. Further experiments to study the effect of EA on the PI3K/AKT/mTOR signaling pathway by immunohistochemistry and WB analysis reveal that the levels of p-PI3K/PI3K, AKT, p-mTOR/mTOR, and p-p70S6/p70S6 in the injured spinal cord are increased after EA, while the level of PTEN is significantly decreased and caspase 3, closely related to cell apoptosis, is also decreased.

## 5. Conclusion

Collectively, EA has an excellent therapeutic effect on SCI, by which the results suggest that EA on GV14 and GV4 could improve the recovery of locomotor function and histological morphology change after SCI in rats, through the PI3K/AKT/mTOR signaling pathway on cell growth, apoptosis, and autophagy.

## Data Availability

All the data used to support the findings of this study are included within the article.
